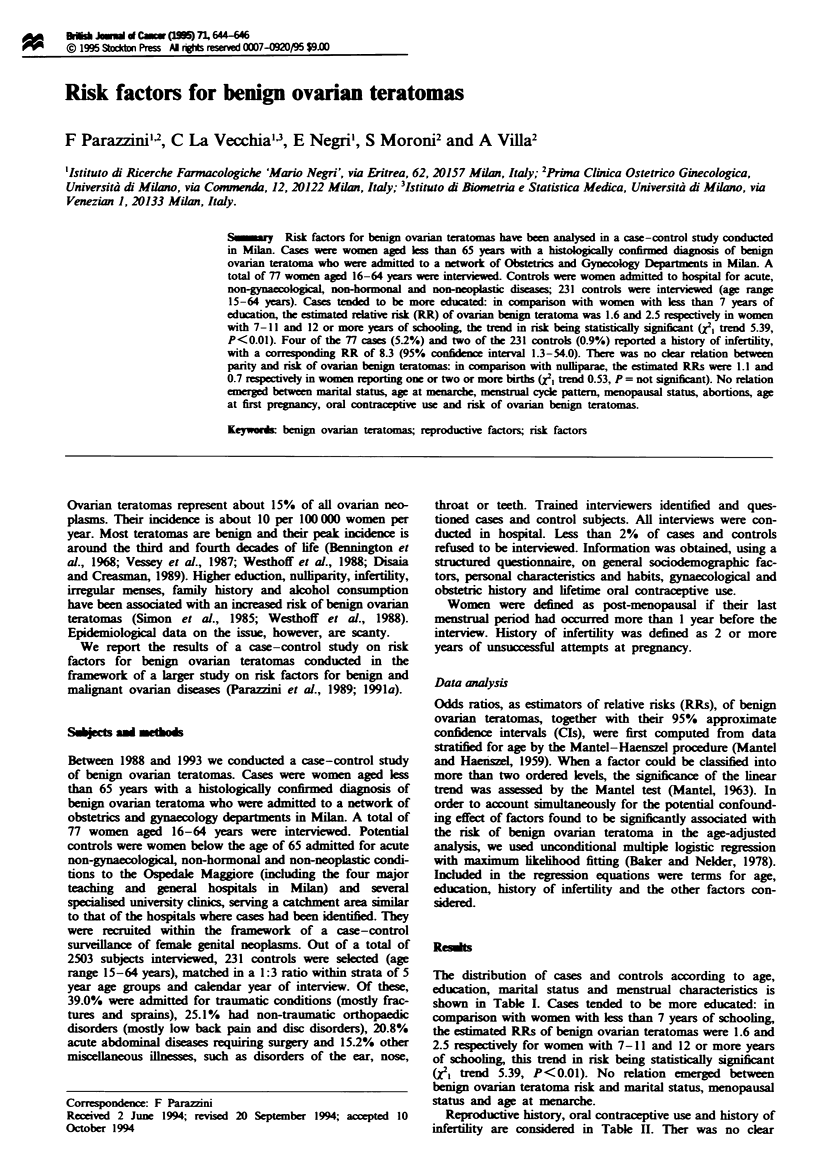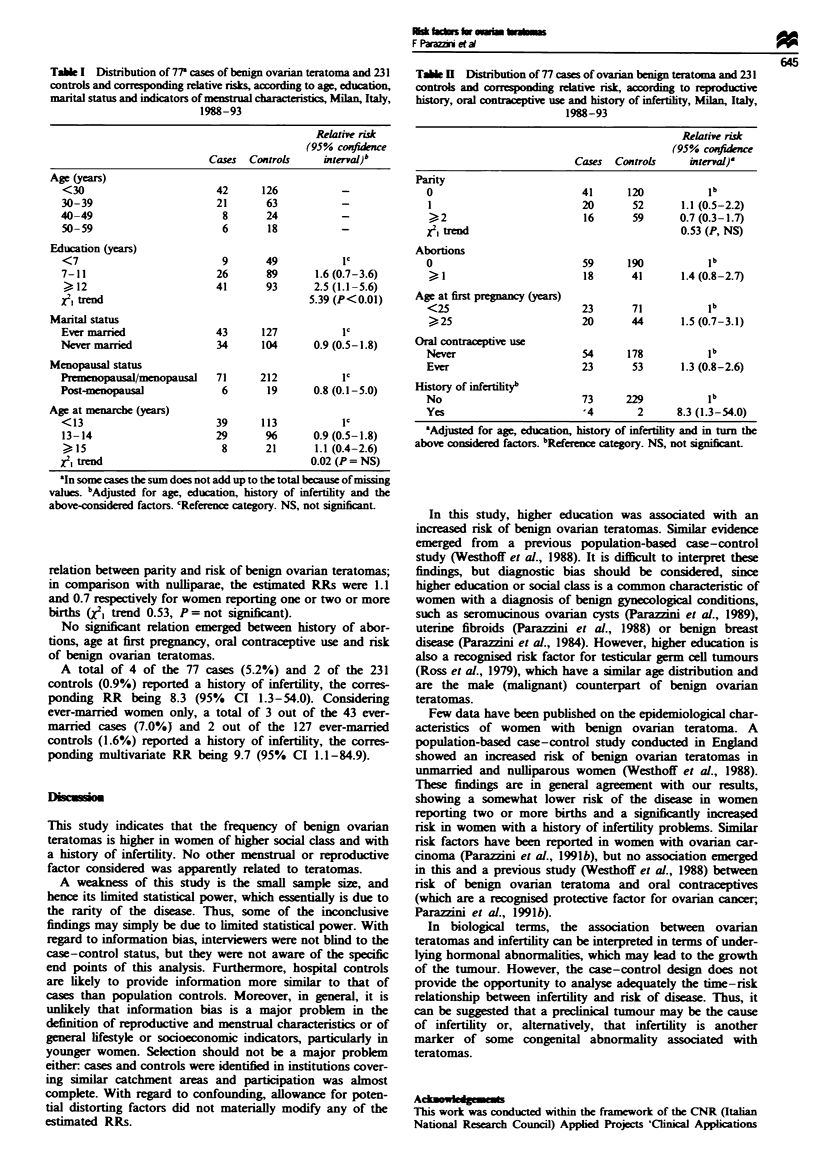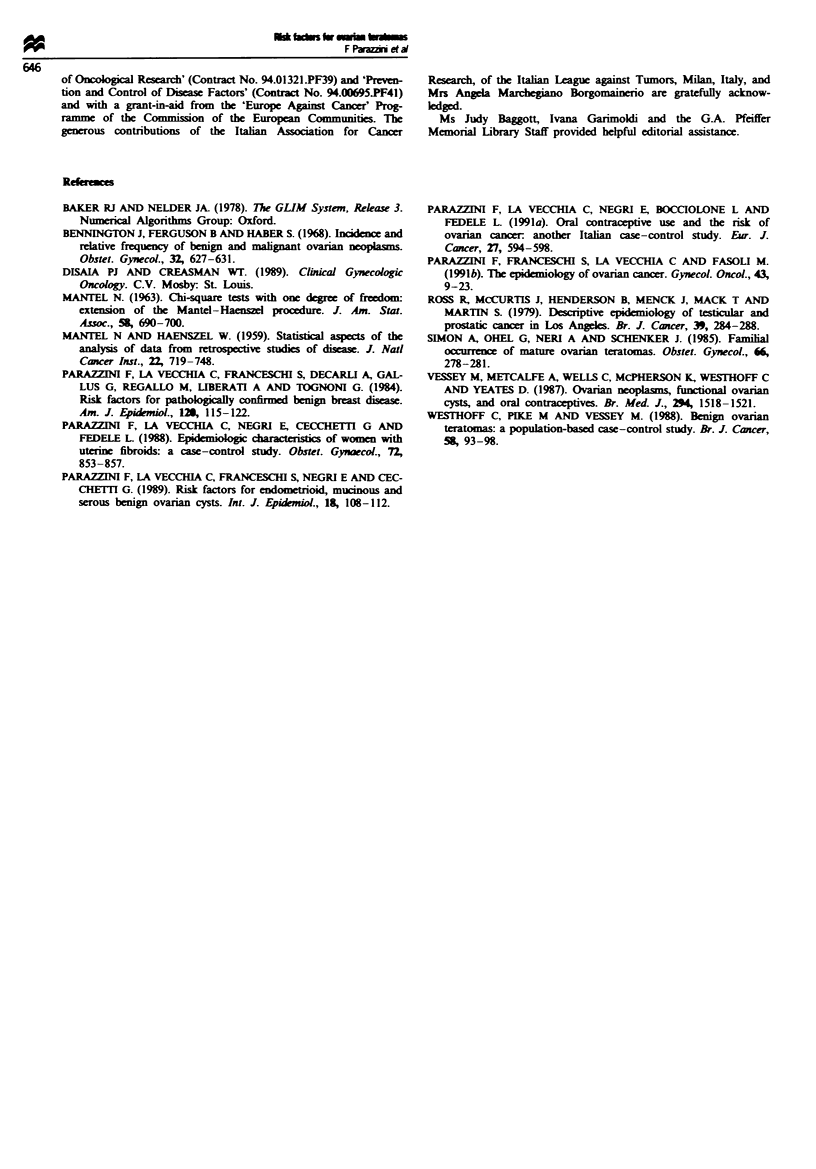# Risk factors for benign ovarian teratomas.

**DOI:** 10.1038/bjc.1995.127

**Published:** 1995-03

**Authors:** F. Parazzini, C. La Vecchia, E. Negri, S. Moroni, A. Villa

**Affiliations:** Istituto di Richerche Farmacologiche Mario Negri, Milan, Italy.

## Abstract

Risk factors for benign ovarian teratomas have been analysed in a case-control study conducted in Milan. Cases were women aged less than 65 years with a histologically confirmed diagnosis of benign ovarian teratoma who were admitted to a network of Obstetrics and Gynecology Departments in Milan. A total of 77 women aged 16-64 years were interviewed. Controls were women admitted to hospital for acute, non-gynaecological, non-hormonal and non-neoplastic diseases; 231 controls were interviewed (age range 15-64 years). Cases tended to be more educated: in comparison with women with less than 7 years of education, the estimated relative risk (RR) of ovarian benign teratoma was 1.6 and 2.5 respectively in women with 7-11 and 12 or more years of schooling, the trend in risk being statistically significant (chi 2(1) trend 5.39, P < 0.01). Four of the 77 cases (5.2%) and two of the 231 controls (0.9%) reported a history of infertility, with a corresponding RR of 8.3 (95% confidence interval 1.3-54.0). There was no clear relation between parity and risk of ovarian benign teratomas: in comparison with nulliparae, the estimated RRs were 1.1 and 0.7 respectively in women reporting one or two or more births (chi 2(1) trend 0.53, P = not significant). No relation emerged between marital status, age at menarche, menstrual cycle pattern, menopausal status, abortions, age at first pregnancy, oral contraceptive use and risk of ovarian benign teratomas.


© 1995 Stockton Press All rights reserved 0007-0920/95 $9.00

### Risk factors for benign ovarian teratomas

F Parazzini[1,2], C La Vecchia[1,3], E Negri[1], S Moroni[2] and A Villa[2]

[1]*Istituto di Ricerche Farmacologiche 'Mario Negri', via Eritrea, 62, 20157 Milan, Italy;* [2]*Prima Clinica Ostetrico Ginecologica, Università di Milano, via Commenda, 12, 20122 Milan, Italy;* [3]*Istituto di Biometria e Statistica Medica, Università di Milano, via Venezian 1, 20133 Milan, Italy.*

**Summary** Risk factors for benign ovarian teratomas have been analysed in a case-control study conducted in Milan. Cases were women aged less than 65 years with a histologically confirmed diagnosis of benign ovarian teratoma who were admitted to a network of Obstetrics and Gynecology Departments in Milan. A total of 77 women aged 16–64 years were interviewed. Controls were women admitted to hospital for acute, non-gynaecological, non-hormonal and non-neoplastic diseases; 231 controls were interviewed (age range 15–64 years). Cases tended to be more educated: in comparison with women with less than 7 years of education, the estimated relative risk (RR) of ovarian benign teratoma was 1.6 and 2.5 respectively in women with 7–11 and 12 or more years of schooling, the trend in risk being statistically significant ($\chi^2_1$ trend 5.39, $P<0.01$). Four of the 77 cases (5.2%) and two of the 231 controls (0.9%) reported a history of infertility, with a corresponding RR of 8.3 (95% confidence interval 1.3–54.0). There was no clear relation between parity and risk of ovarian benign teratomas: in comparison with nulliparae, the estimated RRs were 1.1 and 0.7 respectively in women reporting one or two or more births ($\chi^2_1$ trend 0.53, $P$ = not significant). No relation emerged between marital status, age at menarche, menstrual cycle pattern, menopausal status, abortions, age at first pregnancy, oral contraceptive use and risk of ovarian benign teratomas.

**Keywords:** benign ovarian teratomas; reproductive factors; risk factors

Ovarian teratomas represent about 15% of all ovarian neoplasms. Their incidence is about 10 per 100 000 women per year. Most teratomas are benign and their peak incidence is around the third and fourth decades of life (Bennington *et al.*, 1968; Vessey *et al.*, 1987; Westhoff *et al.*, 1988; Disaia and Creasman, 1989). Higher eduction, nulliparity, infertility, irregular menses, family history and alcohol consumption have been associated with an increased risk of benign ovarian teratomas (Simon *et al.*, 1985; Westhoff *et al.*, 1988). Epidemiological data on the issue, however, are scanty.

We report the results of a case-control study on risk factors for benign ovarian teratomas conducted in the framework of a larger study on risk factors for benign and malignant ovarian diseases (Parazzini *et al.*, 1989; 1991*a*).

### Subjects and methods

Between 1988 and 1993 we conducted a case-control study of benign ovarian teratomas. Cases were women aged less than 65 years with a histologically confirmed diagnosis of benign ovarian teratoma who were admitted to a network of obstetrics and gynaecology departments in Milan. A total of 77 women aged 16–64 years were interviewed. Potential controls were women below the age of 65 admitted for acute non-gynaecological, non-hormonal and non-neoplastic conditions to the Ospedale Maggiore (including the four major teaching and general hospitals in Milan) and several specialised university clinics, serving a catchment area similar to that of the hospitals where cases had been identified. They were recruited within the framework of a case-control surveillance of female genital neoplasms. Out of a total of 2503 subjects interviewed, 231 controls were selected (age range 15–64 years), matched in a 1:3 ratio within strata of 5 year age groups and calendar year of interview. Of these, 39.0% were admitted for traumatic conditions (mostly fractures and sprains), 25.1% had non-traumatic orthopaedic disorders (mostly low back pain and disc disorders), 20.8% acute abdominal diseases requiring surgery and 15.2% other miscellaneous illnesses, such as disorders of the ear, nose, throat or teeth. Trained interviewers identified and questioned cases and control subjects. All interviews were conducted in hospital. Less than 2% of cases and controls refused to be interviewed. Information was obtained, using a structured questionnaire, on general sociodemographic factors, personal characteristics and habits, gynaecological and obstetric history and lifetime oral contraceptive use.

Women were defined as post-menopausal if their last menstrual period had occurred more than 1 year before the interview. History of infertility was defined as 2 or more years of unsuccessful attempts at pregnancy.

### *Data analysis*

Odds ratios, as estimators of relative risks (RRs), of benign ovarian teratomas, together with their 95% approximate confidence intervals (CIs), were first computed from data stratified for age by the Mantel–Haenszel procedure (Mantel and Haenszel, 1959). When a factor could be classified into more than two ordered levels, the significance of the linear trend was assessed by the Mantel test (Mantel, 1963). In order to account simultaneously for the potential confounding effect of factors found to be significantly associated with the risk of benign ovarian teratoma in the age-adjusted analysis, we used unconditional multiple logistic regression with maximum likelihood fitting (Baker and Nelder, 1978). Included in the regression equations were terms for age, education, history of infertility and the other factors considered.

### Results

The distribution of cases and controls according to age, education, marital status and menstrual characteristics is shown in Table I. Cases tended to be more educated: in comparison with women with less than 7 years of schooling, the estimated RRs of benign ovarian teratomas were 1.6 and 2.5 respectively for women with 7–11 and 12 or more years of schooling, this trend in risk being statistically significant ($\chi^2_1$ trend 5.39, $P<0.01$). No relation emerged between benign ovarian teratoma risk and marital status, menopausal status and age at menarche.

Reproductive history, oral contraceptive use and history of infertility are considered in Table II. Ther was no clear

Correspondence: F Parazzini
Received 2 June 1994; revised 20 September 1994; accepted 10 October 1994

**Table I** Distribution of 77[a] cases of benign ovarian teratoma and 231 controls and corresponding relative risks, according to age, education, marital status and indicators of menstrual characteristics, Milan, Italy, 1988–93

| | Cases | Controls | Relative risk (95% confidence interval)[b] |
|---|---|---|---|
| Age (years) | | | |
| <30 | 42 | 126 | – |
| 30–39 | 21 | 63 | – |
| 40–49 | 8 | 24 | – |
| 50–59 | 6 | 18 | – |
| Education (years) | | | |
| <7 | 9 | 49 | 1[c] |
| 7–11 | 26 | 89 | 1.6 (0.7–3.6) |
| ⩾12 | 41 | 93 | 2.5 (1.1–5.6) |
| $\chi^2_1$ trend | | | 5.39 ($P<0.01$) |
| Marital status | | | |
| Ever married | 43 | 127 | 1[c] |
| Never married | 34 | 104 | 0.9 (0.5–1.8) |
| Menopausal status | | | |
| Premenopausal/menopausal | 71 | 212 | 1[c] |
| Post-menopausal | 6 | 19 | 0.8 (0.1–5.0) |
| Age at menarche (years) | | | |
| <13 | 39 | 113 | 1[c] |
| 13–14 | 29 | 96 | 0.9 (0.5–1.8) |
| ⩾15 | 8 | 21 | 1.1 (0.4–2.6) |
| $\chi^2_1$ trend | | | 0.02 ($P$ = NS) |

[a]In some cases the sum does not add up to the total because of missing values. [b]Adjusted for age, education, history of infertility and the above-considered factors. [c]Reference category. NS, not significant.

**Table II** Distribution of 77 cases of ovarian benign teratoma and 231 controls and corresponding relative risk, according to reproductive history, oral contraceptive use and history of infertility, Milan, Italy, 1988–93

| | Cases | Controls | Relative risk (95% confidence interval)[a] |
|---|---|---|---|
| Parity | | | |
| 0 | 41 | 120 | 1[b] |
| 1 | 20 | 52 | 1.1 (0.5–2.2) |
| ⩾2 | 16 | 59 | 0.7 (0.3–1.7) |
| $\chi^2_1$ trend | | | 0.53 ($P$, NS) |
| Abortions | | | |
| 0 | 59 | 190 | 1[b] |
| ⩾1 | 18 | 41 | 1.4 (0.8–2.7) |
| Age at first pregnancy (years) | | | |
| <25 | 23 | 71 | 1[b] |
| ⩾25 | 20 | 44 | 1.5 (0.7–3.1) |
| Oral contraceptive use | | | |
| Never | 54 | 178 | 1[b] |
| Ever | 23 | 53 | 1.3 (0.8–2.6) |
| History of infertility[b] | | | |
| No | 73 | 229 | 1[b] |
| Yes | 4 | 2 | 8.3 (1.3–54.0) |

[a]Adjusted for age, education, history of infertility and in turn the above considered factors. [b]Reference category. NS, not significant.

relation between parity and risk of benign ovarian teratomas; in comparison with nulliparae, the estimated RRs were 1.1 and 0.7 respectively for women reporting one or two or more births ($\chi^2_1$ trend 0.53, $P$ = not significant).

No significant relation emerged between history of abortions, age at first pregnancy, oral contraceptive use and risk of benign ovarian teratomas.

A total of 4 of the 77 cases (5.2%) and 2 of the 231 controls (0.9%) reported a history of infertility, the corresponding RR being 8.3 (95% CI 1.3–54.0). Considering ever-married women only, a total of 3 out of the 43 ever-married cases (7.0%) and 2 out of the 127 ever-married controls (1.6%) reported a history of infertility, the corresponding multivariate RR being 9.7 (95% CI 1.1–84.9).

### Discussion

This study indicates that the frequency of benign ovarian teratomas is higher in women of higher social class and with a history of infertility. No other menstrual or reproductive factor considered was apparently related to teratomas.

A weakness of this study is the small sample size, and hence its limited statistical power, which essentially is due to the rarity of the disease. Thus, some of the inconclusive findings may simply be due to limited statistical power. With regard to information bias, interviewers were not blind to the case–control status, but they were not aware of the specific end points of this analysis. Furthermore, hospital controls are likely to provide information more similar to that of cases than population controls. Moreover, in general, it is unlikely that information bias is a major problem in the definition of reproductive and menstrual characteristics or of general lifestyle or socioeconomic indicators, particularly in younger women. Selection should not be a major problem either: cases and controls were identified in institutions covering similar catchment areas and participation was almost complete. With regard to confounding, allowance for potential distorting factors did not materially modify any of the estimated RRs.

In this study, higher education was associated with an increased risk of benign ovarian teratomas. Similar evidence emerged from a previous population-based case–control study (Westhoff *et al.*, 1988). It is difficult to interpret these findings, but diagnostic bias should be considered, since higher education or social class is a common characteristic of women with a diagnosis of benign gynecological conditions, such as seromucinous ovarian cysts (Parazzini *et al.*, 1989), uterine fibroids (Parazzini *et al.*, 1988) or benign breast disease (Parazzini *et al.*, 1984). However, higher education is also a recognised risk factor for testicular germ cell tumours (Ross *et al.*, 1979), which have a similar age distribution and are the male (malignant) counterpart of benign ovarian teratomas.

Few data have been published on the epidemiological characteristics of women with benign ovarian teratoma. A population-based case–control study conducted in England showed an increased risk of benign ovarian teratomas in unmarried and nulliparous women (Westhoff *et al.*, 1988). These findings are in general agreement with our results, showing a somewhat lower risk of the disease in women reporting two or more births and a significantly increased risk in women with a history of infertility problems. Similar risk factors have been reported in women with ovarian carcinoma (Parazzini *et al.*, 1991*b*), but no association emerged in this and a previous study (Westhoff *et al.*, 1988) between risk of benign ovarian teratoma and oral contraceptives (which are a recognised protective factor for ovarian cancer; Parazzini *et al.*, 1991*b*).

In biological terms, the association between ovarian teratomas and infertility can be interpreted in terms of underlying hormonal abnormalities, which may lead to the growth of the tumour. However, the case–control design does not provide the opportunity to analyse adequately the time–risk relationship between infertility and risk of disease. Thus, it can be suggested that a preclinical tumour may be the cause of infertility or, alternatively, that infertility is another marker of some congenital abnormality associated with teratomas.

**Acknowledgements**
This work was conducted within the framework of the CNR (Italian National Research Council) Applied Projects 'Clinical Applications of Oncological Research' (Contract No. 94.01321.PF39) and 'Prevention and Control of Disease Factors' (Contract No. 94.00695.PF41) and with a grant-in-aid from the 'Europe Against Cancer' Programme of the Commission of the European Communities. The generous contributions of the Italian Association for Cancer Research, of the Italian League against Tumors, Milan, Italy, and Mrs Angela Marchegiano Borgomainerio are gratefully acknowledged.

Ms Judy Baggott, Ivana Garimoldi and the G.A. Pfeiffer Memorial Library Staff provided helpful editorial assistance.

### References

BAKER RJ AND NELDER JA. (1978). *The GLIM System, Release 3.* Numerical Algorithms Group: Oxford.

BENNINGTON J, FERGUSON B AND HABER S. (1968). Incidence and relative frequency of benign and malignant ovarian neoplasms. *Obstet. Gynecol.*, **32**, 627–631.

DISAIA PJ AND CREASMAN WT. (1989). *Clinical Gynecologic Oncology*. C.V. Mosby: St. Louis.

MANTEL N. (1963). Chi-square tests with one degree of freedom: extension of the Mantel–Haenszel procedure. *J. Am. Stat. Assoc.*, **58**, 690–700.

MANTEL N AND HAENSZEL W. (1959). Statistical aspects of the analysis of data from retrospective studies of disease. *J. Natl Cancer Inst.*, **22**, 719–748.

PARAZZINI F, LA VECCHIA C, FRANCESCHI S, DECARLI A, GALLUS G, REGALLO M, LIBERATI A AND TOGNONI G. (1984). Risk factors for pathologically confirmed benign breast disease. *Am. J. Epidemiol.*, **120**, 115–122.

PARAZZINI F, LA VECCHIA C, NEGRI E, CECCHETTI G AND FEDELE L. (1988). Epidemiologic characteristics of women with uterine fibroids: a case–control study. *Obstet. Gynaecol.*, **72**, 853–857.

PARAZZINI F, LA VECCHIA C, FRANCESCHI S, NEGRI E AND CECCHETTI G. (1989). Risk factors for endometrioid, mucinous and serous benign ovarian cysts. *Int. J. Epidemiol.*, **18**, 108–112.

PARAZZINI F, LA VECCHIA C, NEGRI E, BOCCIOLONE L AND FEDELE L. (1991*a*). Oral contraceptive use and the risk of ovarian cancer: another Italian case–control study. *Eur. J. Cancer*, **27**, 594–598.

PARAZZINI F, FRANCESCHI S, LA VECCHIA C AND FASOLI M. (1991*b*). The epidemiology of ovarian cancer. *Gynecol. Oncol.*, **43**, 9–23.

ROSS R, McCURTIS J, HENDERSON B, MENCK J, MACK T AND MARTIN S. (1979). Descriptive epidemiology of testicular and prostatic cancer in Los Angeles. *Br. J. Cancer*, **39**, 284–288.

SIMON A, OHEL G, NERI A AND SCHENKER J. (1985). Familial occurrence of mature ovarian teratomas. *Obstet. Gynecol.*, **66**, 278–281.

VESSEY M, METCALFE A, WELLS C, McPHERSON K, WESTHOFF C AND YEATES D. (1987). Ovarian neoplasms, functional ovarian cysts, and oral contraceptives. *Br. Med. J.*, **294**, 1518–1521.

WESTHOFF C, PIKE M AND VESSEY M. (1988). Benign ovarian teratomas: a population-based case–control study. *Br. J. Cancer*, **58**, 93–98.